# MYB3R-SCL28-SMR module with a role in cell size control negatively regulates G2 progression in *Arabidopsis*

**DOI:** 10.1080/15592324.2022.2153209

**Published:** 2022-12-28

**Authors:** Hirotomo Takatsuka, Yuji Nomoto, Kesuke Yamada, Keito Mineta, Christian Breuer, Takashi Ishida, Ayumi Yamagami, Keiko Sugimoto, Takeshi Nakano, Masaki Ito

**Affiliations:** a School of Biological Science and Technology, College of Science and Engineering, Kanazawa University, Kakuma-machi, Kanazawa, 920-1192, Japan; b RIKEN Center for Sustainable Resource Science, Yokohama, 230-0045, Japan; c Faculty of Advanced Science and Technology, Kumamoto University, Kumamoto, 860-8555, Japan; d Graduate School of Biostudies, Kyoto University, Kyoto, Japan; e Gene Discovery Research Group, RIKEN Center for Sustainable Resource Science, Tsukuba, Japan; f Department of Biological Sciences, Graduate School of Science, the University of Tokyo, 7-3-1 Hongo, Bunkyo-ku, Tokyo 113-0033, Japan; g School of Engineering and Applied Sciences, National University of Mongolia, Ulaanbaatar, Mongolia

**Keywords:** Cell cycle, G2 phase, cell size, transcription factor, GRAS family, MYB3Rs

## Abstract

Cell size control is one of the prerequisites for plant growth and development. Recently, a GRAS family transcription factor, SCARECROW-LIKE28 (SCL28), was identified as a critical regulator for both mitotic and postmitotic cell-size control. Here, we show that *SCL28* is specifically expressed in proliferating cells and exerts its function to delay G2 progression during mitotic cell cycle in *Arabidopsis thaliana*. Overexpression of *SCL28* provokes a significant enlargement of cells in various organs and tissues, such as leaves, flowers and seeds, to different extents depending on the type of cells. The increased cell size is most likely due to a delayed G2 progression and accelerated onset of endoreplication, an atypical cell cycle repeating DNA replication without cytokinesis or mitosis. Unlike *DWARF AND LOW-TILLERING*, a rice ortholog of *SCL28*, SCL28 may not have a role in brassinosteroid (BR) signaling because sensitivity against brassinazole, a BR biosynthesis inhibitor, was not dramatically altered in *scl28* mutant and *SCL28*-overexpressing plants. Collectively, our findings strengthen a recently proposed model of cell size control by SCL28 and suggest the presence of diversified evolutionary mechanisms for the regulation and action of SCL28.

## Introduction

As cell cycle progression greatly impacts both cell size and number, it needs to be strictly controlled in multicellular organisms. In plants, the cell cycle is largely divided into two modes, i.e., mitotic division cycle and endocycle (also known as endoreplication).^1,2^ The mitotic cycle, which exclusively occurs in meristematic cells, is composed of four distinct phases: Gap1 (G1), DNA synthesis (S), Gap2 (G2), and mitosis (M). In many plant species, including *Arabidopsis thaliana*, after leaving the meristem, cells exit proliferation and instead initiate endoreplication, a specialized cell cycle mode in which cells repeat DNA replication without cytokinesis or mitosis, thereby leading to an increase in DNA content and cell enlargement.^1,2^ It has been proposed that the key step for the transition from mitotic cycle to endocycle lies within the G2 phase, in which cells either proceed into the M phase or skip mitosis and enter the next cycle.^3,4^ Therefore, cell cycle regulation at G2 is critical in deciding whether to divide and increase cell number or initiate endoreplication and increase cell size.

Previously, we identified MYB3R transcription factors acting as central regulators of G2/M progression.^5–8^ The *Arabidopsis* genome has five MYB3R genes, which are further divided into two main subtypes with opposing functions: the transcriptional activators MYB3R1 and MYB3R4,^6,7^ and the transcriptional repressors MYB3R3 and MYB3R5.^8^ MYB3Rs control G2/M-specific genes, such as *CYCLIN B1;1* (*CYCB1;1), CYCLIN-DEPENDENT KINASE B2* (*CDKB2*), and *KNOLLE* (*KN*), all of which have a promotive role in G2/M progression.^7,8^ Recently, we discovered another important direct target of MYB3Rs, namely, *SCARECROW-LIKE28* (*SCL28*), which encodes for a member of the GRAS family transcription factors.^4^ Counterintuitively, as proved by the shorter and longer cell cycles detected in *scl28* knockout mutants and *SCL28*-overexpressing plants, respectively, *SCL28* harms cell cycle progression, despite being directly induced by MYB3R4, whose main function is to accelerate G2/M progression.^4^ Our analyses, integrating cytological, genetic, and biochemical approaches, revealed that SCL28 forms a dimer with the AP2-type transcription factor AtSMOS1. This directly up-regulates the expression of a subset of *SIAMESE-RELATED* (*SMR*) family genes that encode plant-specific inhibitors of cyclin-dependent kinases, thereby delaying G2/M progression and triggering the transition from mitotic cycle to endocycle.^4^

Here, we analyzed the function and expression of *SCL28* in cell types that have not been investigated in our previous research. We showed that the effect of SCL28 on cell size was widely detected in all cell types examined in this study, although its impact largely depended on types of cells. The results presented here reinforce the recently proposed notion that a hierarchical transcriptional network consisting of MYB3Rs-SCL28-SMRs provides a universal framework to adjust cell size and number and ultimately ensure robust organ growth in plants.

## Results & Discussion

**Figure 1.**
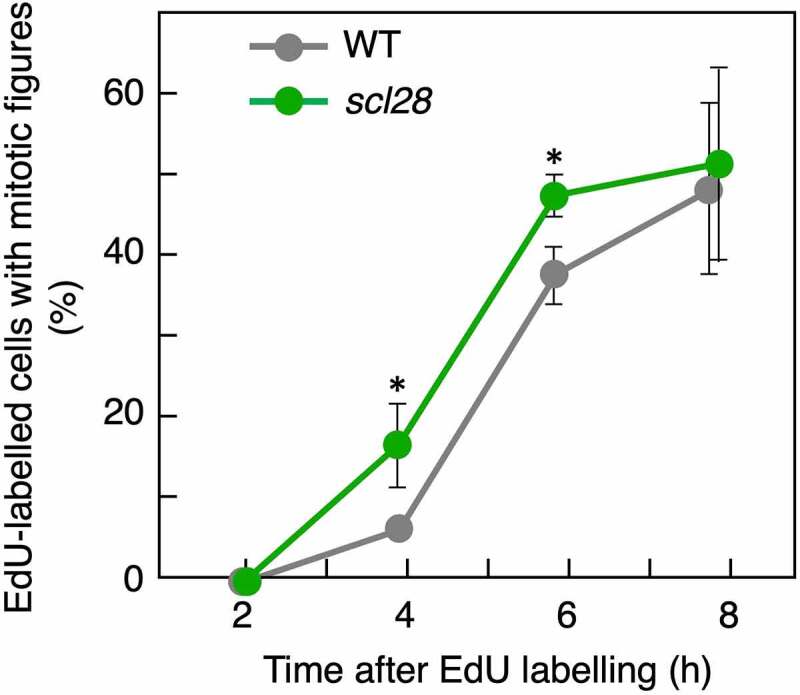
G2 phase progression is accelerated in *scl28*. Analysis of the G2 phase duration in meristematic cells. Five-day-old seedlings of wild-type (WT) and *scl28* were pulse-labeled with EdU for 15 min, transferred back to MS solid medium, and collected 2, 4, 6 and 8 h after transfer. Root tips were double-stained with EdU and DAPI, and the meristematic epidermal cells with mitotic figures were counted. The percentages of EdU-positive cells among those showing mitotic figures were calculated, and data are presented as mean ± SD (*n* = 3). Significant differences were determined using the Student’s test (*P* < .05).

Our recent cell cycle analysis employing the PROLIFERATING CELLULAR NUCLEAR ANTIGEN (PCNA)-GFP reporter revealed that the G2/M phase is shorter in *scl28* knockout mutants.^4^ Here, focusing more specifically on the role of SCL28 in G2 progression, we applied a recently developed method using 5-ethynyl-2’-deoxyuridine (EdU), which is exclusively incorporated into replicating chromosomes in S-phase cells.^9^ After incubation with EdU for 15 min, EdU-labeled root meristematic cells pass through the G2 phase and eventually enter the M phase showing mitotic figures with the EdU signal.^9^ In principle, in a cell population with a shorter G2 duration, it can be expected that EdU-positive mitotic cells should appear earlier after EdU labeling, and that their frequency should increase more steeply over time. In wild-type roots, EdU-labeled cells with mitotic figures first appeared at 4 h after pulse labeling with EdU and gradually increased thereafter (Figure 1), implying that the cells took at least 4 h to pass through the entire G2 phase period and enter the M phase. On the other hand, we detected the highest percentages of EdU-positive mitotic cells in *scl28* from 4 h onward (Figure 1), which is indicative of the accelerated G2 progression in *scl28*. Therefore, this result validates our recent report suggesting that SCL28 acts as a negative regulator of G2 progression in proliferating root cells.^4^

**Figure 2.**
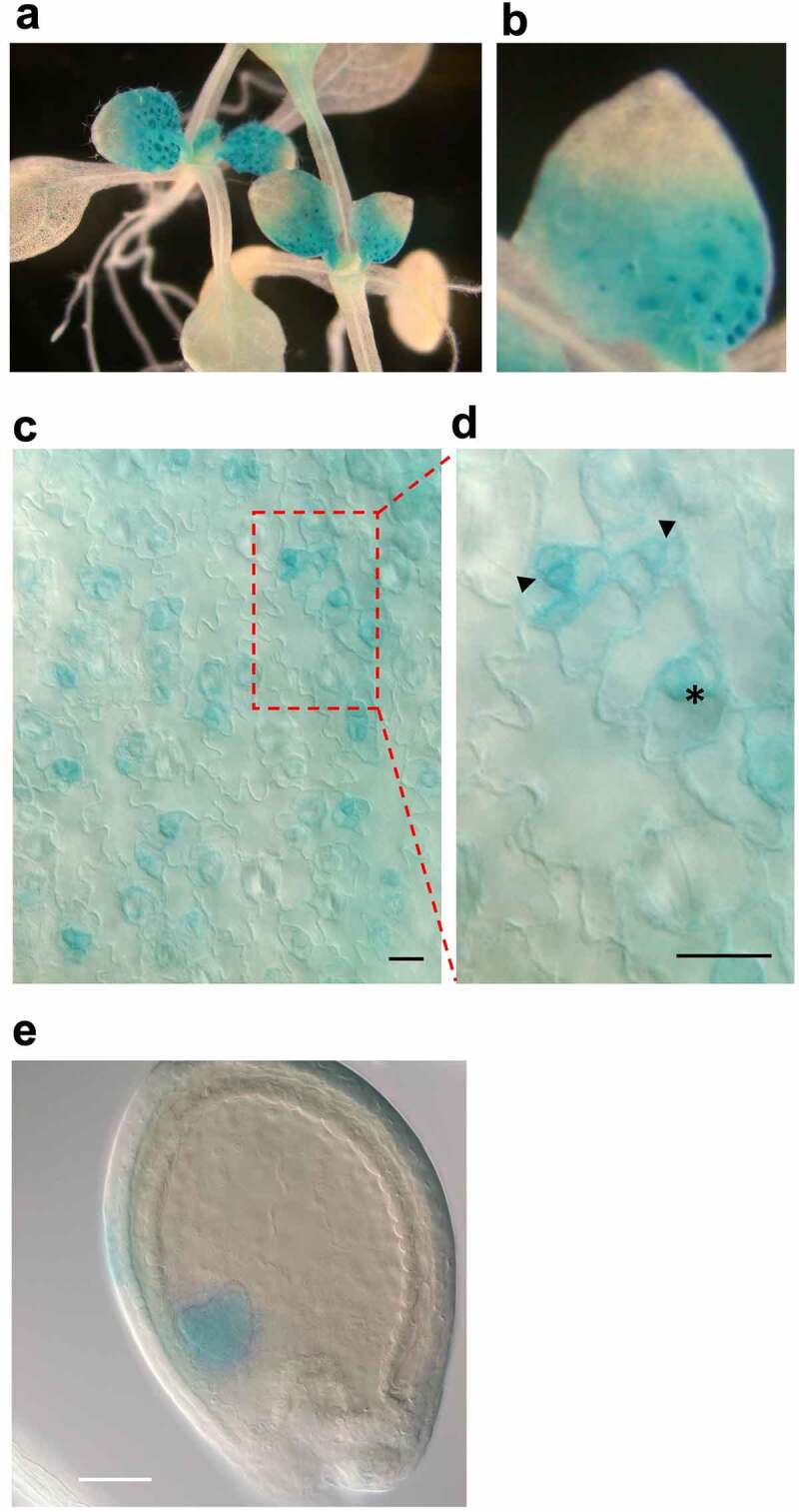
*SCL28* expression is confined to proliferating cells. GUS staining of transgenic plants harboring *pSCL28:GUS*. (a and b): Shoots (a) and fourth leaf (b) of a GUS-stained seedling. (c and d): Leaf epidermis. The arrowheads and asterisk indicate meristemoids and guard mother cells, respectively. Scale bars = 20 µm. (e) Heart-stage embryo. Scale bar = 100 µm.

The *SCL28* promoter is active in proliferating cells in both shoot and root apical meristems.^4^ However, the expression of *SCL28* in other organs containing proliferating cells has not been examined yet. Thus, we conducted an expression analysis of the *SCL28* gene with the reporter fusion where the *SCL28* promoter fused to the GUS gene (hereafter referred to as *pSCL28:GUS*) in leaves and seeds (Figure 2). GUS staining was observed in young developing leaves, but not in cotyledons and fully developed leaves in which most cells already exit proliferation (Figure 2a). In developing leaves, *pSCL28:GUS* expression was confined to the leaf base, where cells continue to proliferate longer than other leaf areas (Figure 2b). The observed expression pattern of *SCL28* resembles that reported for B-type cyclin *CYCB1;1*, whose transcripts specifically accumulate during the G2/M phase,^10^ suggesting that *SCL28* is expressed in the mitotically active cells in leaves. A closer observation of the leaf epidermis revealed stronger GUS staining in proliferating stomatal precursor cells, such as meristemoids and guard mother cells, further confirming that *SCL28* expression is specific to proliferating cells (Figure 2c, d). In young ovules, GUS signals were observed in developing embryos with active cell division, but not in seed coats in which cells already exit proliferation at this stage (Figure 2e). Combined with our previous research,^4^ these results demonstrate that *SCL28* is specifically expressed in the proliferating cells of developing organs.

**Figure 3.**
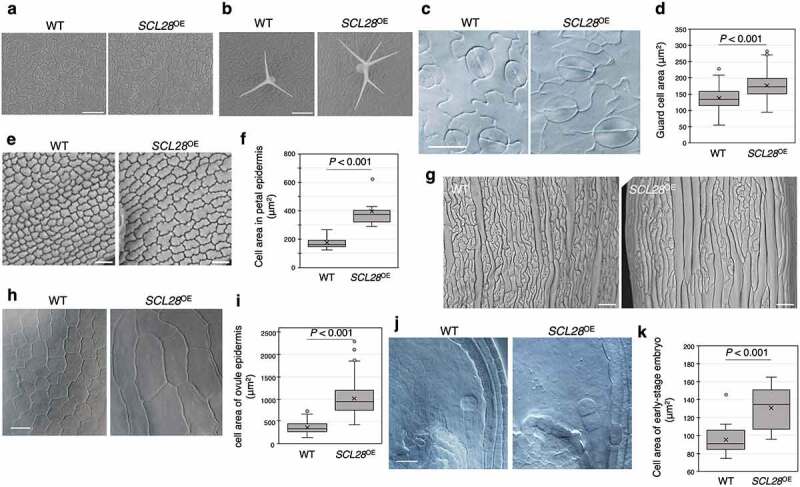
*SCL28* overexpression induces cell enlargement. Phenotypes of *SCL28*-overexpressing plants. (a) Leaf epidermis of wild type (WT) and *SCL28*^OE^ seedlings at 20 day after germination (DAG). (b) Trichomes on the third leaves of WT and *SCL28*^OE^ seedlings at 20 DAG. (c) Guard cells in abaxial epidermis of 1st leaf pair from WT and *SCL28*^OE^ seedlings at 12 DAG. (d) Quantitative measurement of guard cell size. Only mature guard cells possessing recognizable pores were analyzed (*n* ≥ 160). (e) Cells in abaxial epidermis of petals from WT and *SCL28*^OE^ plants. (f) Quantitative measurement of cell size in petal epidermis. Epidermal cells at apical region of petal were analyzed (*n* = 10). (g) Cells in abaxial epidermis of sepals from WT and *SCL28*^OE^ plants. (h) Epidermal cells of ovules from WT and *SCL28*^OE^ plants. (i) Quantitative measurement of cell size in ovule epidermis (*n* ≥ 130). (j) Cells of 2-cell-stage embryo developed in WT and *SCL28*^OE^ plants. (k) Quantitative measurement of size of embryo cells at 2-cell stage (*n* ≥ 20). In (a), (b), (e), and (g), plant samples were observed with scanning electron microscopy. In (c), (h), and (j), plant samples were cleared and observed with differential interference contrast (DIC) microscopy. Scale bars indicate 100 µm (a), 150 µm (b), 20 µm (c, h and j), 25 µm (e), and 50 µm (g).

We then examined the effect of *SCL28* overexpression on cell size in various types of cells in different organs, which we have not extensively analyzed previously^4^ (Figure 3). We used transgenic plants overexpressing *SCL28* under the control of the *RIBOSOMAL PROTEIN 5A* (*RPS5A*) promoter (hereafter called *SCL28*^OE^), which was shown to display a increased cell size in various tissues and organs in our previous study.^4^ In leaves, we confirmed significant enlargement of pavement cells as observed using scanning electron microscope (SEM) (Figure 3a). Consistent with increased cellular policy in *SCL28*^OE^ leaves,^4^ an increase in trichome branch number, a typical sign of enhanced endoreplication,^11^ was frequently observed in *SCL28*^OE^ leaves (Figure 3b). In addition, small difference in guard cell size between wild type and SCL28^OE^ plants was found to be significant after quantitative measurement (Figure 3c, d). We also observed significantly increased size of epidermal cells in various organs such as petals (Figure 3e, f), sepals (Figure 3g), and ovules (Figure 3h, i). Interestingly, in developing embryo, cells are already significantly enlarged as early as 2-cell stage. (Figure 3j, k). These results imply that SCL28-induced G2 delay may affect cell size both during proliferation and after terminal differentiation. Increased size of terminally-differentiated cells may be contributed by the premature transition from the mitotic cycle to endoreplication, which may induce increased cellular ploidy levels. It is also worth mentioning that the extent of cell enlargement by *SCL28*^OE^ varied significantly depending on the type of cells, tissues, and organs, leading us to hypothesize that cell size is also regulated by cell type-specific mechanisms that do not involve SCL28. However, this argument should await further careful investigation because the effect of SCL28 may be influenced by expression levels of SCL28 which may vary spatially and temporally in *SCL28*^OE^ plants.

**Figure 4.**
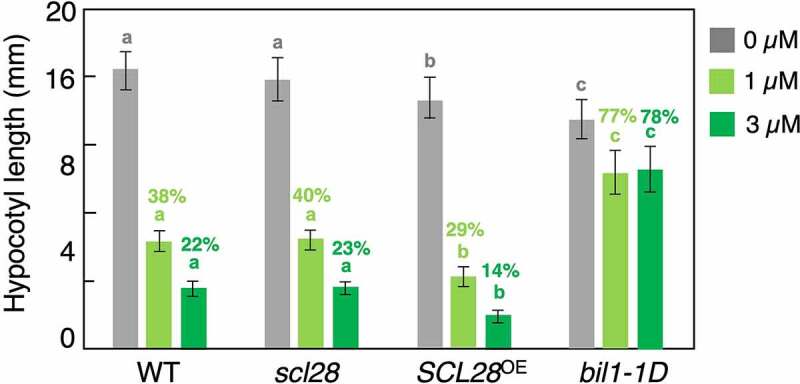
SCL28 is not involved in brassinosteroid signaling. Hypocotyl length of wild-type (WT), *scl28, SCL28^OE^* and *bil1-1D* grown on medium either with or without Brz in the dark for 7 days. Data are presented as mean ± SD (*n* = 30). Bars with different letters indicate significant differences, as revealed by Tukey’s test (*P* < .05). Figures above each bar indicate the relative hypocotyl length of Brz-treated plants compared with that of control plants (set to 100%) for each genotype.

The *SCL28* gene is evolutionarily conserved across plant species, among which a rice ortholog of *SCL28, DWARF AND LOW-TILLERING* (*DLT*), has been the most extensively studied. Similarly to *scl28* in *Arabidopsis*, various organs of the *dlt* mutant are composed of aberrantly small cells,^12^ suggesting that the role of *SCL28* homologs in cell size control might be conserved between *Arabidopsis* and rice. The *DLT* gene was originally identified as a signaling component involved in brassinosteroid (BR) responses in rice;^12^ however, whether *Arabidopsis* SCL28 participates in BR signaling remains unclear. To address this issue, we conducted the hypocotyl growth assay, which has been widely used to assess the BR response^13,14^ (Figure 4). In the presence of brassinazole (Brz), a BR synthesis inhibitor, hypocotyl elongation of dark-grown wild-type seedlings was inhibited in a dose-dependent manner. In contrast, the dominant-negative mutants of *BRZ-INSENSITIVE-LONG HYPOCOTYL 1* (*BIL1*)/*BRASSINAZOLE-RESISTANT 1* (*BZR1*), which is a master transcription factor of BR signaling, exhibited a phenotype that was more tolerant to Brz-induced growth inhibition in hypocotyls, consistent with previous studies.^13^ In both *scl28* knockout mutants and *SCL28*-overexpressing plants, hypocotyl elongation was inhibited to a similar extent as in wild-type plants, suggesting that SCL28, unlike DLT in rice, may not play a role in BR signaling in *Arabidopsis* (Figure 4). It is unknown whether *DLT* is transcriptionally regulated by MYB3Rs and specifically expressed during G2/M in the rice cell cycle. More importantly, it remains to be explored whether cell size control by DLT is exclusively mediated by BR signaling or it is also mediated by the transcriptional activation of *SMR* family genes in rice. Studying in more detail rice DLT and SCL28 orthologs in other plant species would reveal evolutionary aspects of the MYB3R-SCL28-SMR pathway and how this module emerged during plant evolution.

**Figure 5:**
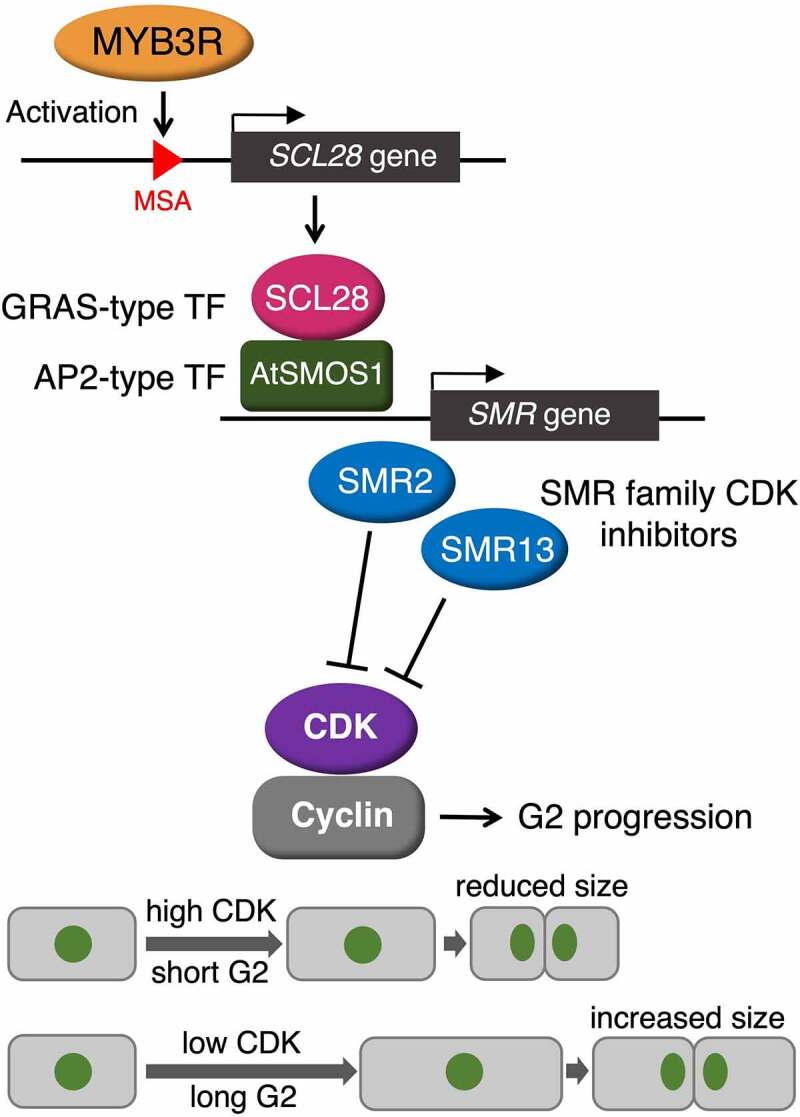
Schematic diagram for regulation and action of SCL28. A GRAS-type transcription factor, SCL28, is expressed under control of MYB3R transcription factors, and interacts with an AP2-type transcription factor called AtSMOS1, forming active heterodimer, which, in turn, activates transcription of SMR family genes, such as *SMR2* and *SMR13*, encoding CDK inhibitors. Increased SMR expression in proliferating cells induces decreased CDK activity and prolonged G2 duration in the cell cycle. Action of SCL28, therefore, modifies CDK activity and G2 duration, thereby affecting positively size of proliferating cells, which is known to be determined by a balance between cell cycle duration and cell growth rate.

Plant production relies largely on plant organ growth, which is, in principle, determined by the size and number of component cells. Therefore, our discovery of SCL28 with a strong positive effect on cell size would be potentially useful for improving crop yield and generation of plants with higher biomass. However, moderate expression changes of SCL28 quantitatively modified cell size but also influenced cell number in an opposing manner, so that total organ size was not dramatically changed in *Arabidopsis*. The observed trade-off between cell size and number may be caused by the dual role of SCL28 in both cell proliferation and postmitotic cell expansion. Alternatively, it may be due to a general mechanism called compensation, which explains the activation of postmitotic cell expansion when cell proliferation is inhibited during leaf development.^15,16^ Elucidating the unsolved mechanisms behind the general trade-off between size and number caused by modified SCL28 expression may thus help us increase plant growth and production, allowing a sustainable agricultural production of food, bioenergy, and biomaterials.

In conclusion, this study provided further strong evidence that SCL28 is specifically expressed in proliferating cells in a wide variety of developing organs and inhibits G2 progression in the mitotic cell cycle. All our present and previous data support our view that the MYB3R-SCL28-SMR module plays an important role in cell size control through negative cell cycle regulation at G2 (Figure 5). A better understanding of the action of SCL28 on cell size control would help reveal the hidden mechanism underlying the general trade-off between cell size and number, which often interferes with genetic engineering projects aiming to generate high-biomass plants, and allow sustainable production of food and bioenergy in the future.

## Materials and methods

***Plant materials and growth condition*** *Arabidopsis thaliana* Columbia (Col) was used as the wild-type plant. pSCL28:GUS, *SCL28*^OE^ (pRPS5A:SCL28), *bil1-1D*, and *scl28* lines were described previously.^4,17^ The methods for seed sterilization and the conditions for plant growth have been previously described.^4^ Briefly, plants were grown on half-strength Murashige and Skoog (1/2MS) medium containing 2% sucrose and 1.0% agar (Wako) at 22°C under continuous light condition. For hypocotyle elongation assay, plants were grown on 1/2MS medium either with or without Brz in the dark for 7 days.

***Phenotype analysis*** GUS staining and clearing of plant samples were described previously.^4^ To observe epidermal cells of leaves, petals and sepals, detached plant organs were directly examined under a TM-3000 table-top scanning electron microscope (Hitachi) equipped with a cooling stage. EdU labelling of elongating roots, double staining with EdU and 4’,6-diamidino-2-phenylindole (DAPI), and conting labelled mitoic cells were conducted as described previously.^18^ Quantification of cell size were performed with Fiji software using images of plant tissues obtained by SEM or DIC observations. Data was analyzed statistically with either Student’s test or Tukey’s test.
